# DNA Hydroxymethylation at the Interface of the Environment and Nonalcoholic Fatty Liver Disease

**DOI:** 10.3390/ijerph16152791

**Published:** 2019-08-05

**Authors:** Stella Tommasi, Ahmad Besaratinia

**Affiliations:** Department of Preventive Medicine, Keck School of Medicine, University of Southern California, M/C 9603, Los Angeles, CA 90033, USA

**Keywords:** epigenetics, xenobiotics, NAFLD, steatosis, oxidative stress

## Abstract

Non-alcoholic fatty liver disease (NAFLD) is one of the most prevalent forms of chronic liver disorders among adults, children, and adolescents, and a growing epidemic, worldwide. Notwithstanding the known susceptibility factors for NAFLD, i.e., obesity and metabolic syndrome, the exact cause(s) of this disease and the underlying mechanisms of its initiation and progression are not fully elucidated. NAFLD is a multi-faceted disease with metabolic, genetic, epigenetic, and environmental determinants. Accumulating evidence shows that exposure to environmental toxicants contributes to the development of NAFLD by promoting mitochondrial dysfunction and generating reactive oxygen species in the liver. Imbalances in the redox state of the cells are known to cause alterations in the patterns of 5-hydroxymethylcytosine (5hmC), the oxidative product of 5-methylcytosine (5mC), thereby influencing gene regulation. The 5hmC-mediated deregulation of genes involved in hepatic metabolism is an emerging area of research in NAFLD. This review summarizes our current knowledge on the interactive role of xenobiotic exposure and DNA hydroxymethylation in the pathogenesis of fatty liver disease. Increasing the mechanistic knowledge of NAFLD initiation and progression is crucial for the development of new and effective strategies for prevention and treatment of this disease.

## 1. Introduction

Nonalcoholic fatty liver disease (NAFLD) is the most common cause of chronic liver disease worldwide, affecting 25% of the global population [1,2,3,4,5,6]. The prevalence of NAFLD is rising in many parts of the world, especially in developed countries [7]. In the United States alone, between 30 to 40% of the adult population is affected by NAFLD [7]. Among children and adolescents, NAFLD is currently the primary form of liver disease; it is estimated that nearly 10% of the US population aged between two and nineteen has NAFLD [8,9]. NAFLD encompasses a wide spectrum of conditions ranging from benign steatosis, characterized by abnormal lipid accumulation within the hepatocytes, to non-alcoholic steatohepatitis (NASH), a more severe form of liver injury accompanied by inflammation and variable fibrosis. Current trends show that NASH is becoming a major risk factor for cirrhosis and end-stage liver disease requiring transplantation [10,11,12,13].

**Figure 1:**
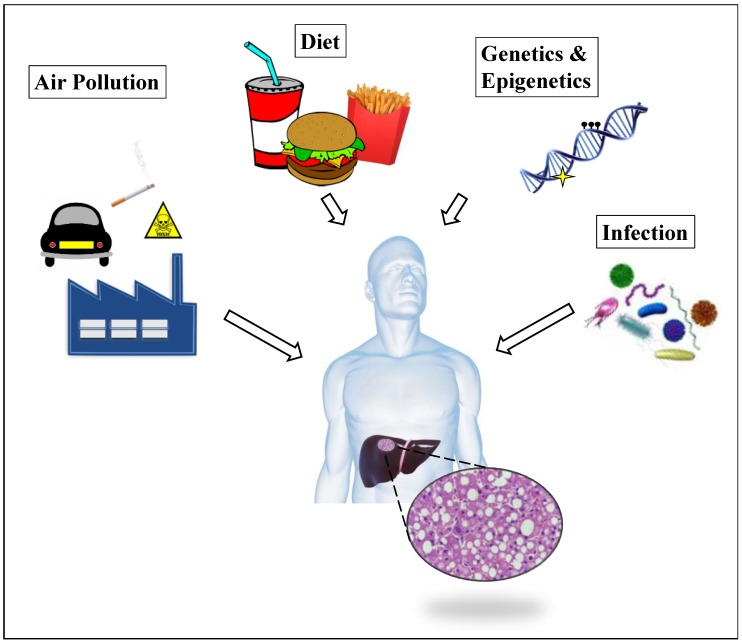
Pathogenesis of NAFLD (non-alcoholic fatty liver disease). NAFLD is characterized by abnormal accumulation of lipids within the hepatocytes (liver steatosis), which manifests with the development of vesicles that can displace the cytoplasm, disrupt cell constituents, and in severe cases, lead to cell rupture/burst. NAFLD is a multi-faceted disease. Multiple factors, including diet and sedentary lifestyle, exposure to toxicants and infectious agents, and genetic and epigenetic mechanisms, are now known to contribute to NAFLD development.

NAFLD is strongly associated with the clinical manifestations of metabolic syndrome, such as obesity, type II diabetes, and dyslipidemia. However, high prevalence of NAFLD does not always correlate with high caloric intake, and non-obese or non-diabetic individuals can also develop the disease [5,6,14]. Although diet and sedentary lifestyle remain the major risk factors for NAFLD, other factors or modifiers, including genetic predisposition, infection, environmental toxicants, and epigenetic mechanisms, may also play a role in the pathogenesis of this disease (Figure 1) [3,5,15,16,17,18]. According to the revised multi-hit hypothesis, a single or combination of risk factors triggers the disruption of lipid homeostasis and fat accumulation that lead to liver steatosis, i.e., first hit. The induced liver steatosis predisposes the affected individual to subsequent hits that can further provoke liver injury through the modulation of pathways involved in mitochondrial dysfunction, oxidative stress, fatty acid biosynthesis, and inflammation, thus giving rise to NASH [15,17]. In other words, the first hit increases an individual’s susceptibility to multiple hits. 

The objective of this review is to outline the impact of environmental exposure on the pathogenesis of NAFLD, with a special focus on an emerging epigenetic mechanism, i.e., DNA hydroxymethylation. Many environmental toxicants are known to promote mitochondrial dysfunction and oxidative stress. There is growing evidence that oxidative stress alters the patterns of 5-hydroxymethylcytosine (5hmC), a primary oxidative product of 5-methylcytosine (5mC), which may influence gene regulation. Thus, investigating the 5hmC-mediated deregulation of genes involved in liver metabolism may provide insight into the interactive role of environment and epigenetics in NAFLD development. A better understanding of the mechanisms of NAFLD genesis and progression can lead to the development of effective preventive strategies and treatment options for this disease. We note that the association between obesity/metabolic syndrome and NAFLD has been discussed in comprehensive reviews [6,14,19]; readers are referred to these elegant references.

## 2. The Contribution of Xenobiotics in the Pathogenesis of NAFLD

The liver plays a crucial role in the maintenance of metabolic homeostasis. Because of its portal location within circulation and its function in the metabolism and excretion of potentially harmful xenobiotics, the liver constitutes the first line of defense against environmental toxicants and contaminants [20]. Liver cells are constantly exposed to significant concentrations of toxic metabolites, making this organ susceptible to chemical- or drug-induced injury (hepatotoxicity). Hepatotoxicity is thus the best indicator of environmental exposure, and manifests in several pathological conditions, with fatty liver or steatosis being the most common and consequential disease [16,21].

An increasing number of studies has shown an association between exposure to xenobiotics, both in the form of pharmaceutical and non-pharmaceutical chemicals, and the pathogenesis of NAFLD [17,22]. Certain environmental contaminants, including perfluoroalkylated substances (PFAS), and polychlorinated biphenyls (PCBs), which are present in a variety of industrial and consumer products, have been shown to promote hepatic steatosis/triglyceride accumulation and liver injury through the generation of reactive oxygen species (ROS) [16,17,18]. Liver steatosis and steatohepatitis are also induced by several pharmaceutical drugs (tetracyclines, tamoxifen, valproic acid, etc.), which are known to interfere with normal lipid metabolism by affecting fatty acid oxidation and retention and mitochondrial function and inducing oxidative stress [17,22].

To date, several animal models have been used to investigate the mechanisms through which environmental chemicals/agents may cause liver steatosis and NAFLD [17,18]. Although each compound may exhibit a different mode of action, the most frequent mechanisms promoted by exposure to environmental chemicals/agents have been mitochondrial dysfunction, impairment of lipid metabolism and excretion, insulin resistance, and elevated cytokine production [23]. Early life exposure of animals to endocrine-disrupting chemicals, including bisphenol A (BPA), benzo[a]pyrene, and phthalates, has been shown to induce fatty liver through mechanisms involving the activation of nuclear hormone receptors and epigenetic alterations [17,18,24].

Few animal studies have also highlighted the role of secondhand smoke (SHS), a known inducer of ROS and oxidative stress [25,26,27], in NAFLD development [28,29,30]. For example, Yuan et al. [30] have shown that sub-chronic exposure of mice to SHS stimulates the synthesis of fatty acids in the liver by modulating two key regulators of lipid metabolism, including AMP-activated kinase (AMPK) and sterol regulatory element binding protein-1c (SREBP-1c). The SHS-exposed mice developed hepatic steatosis, which, according to the authors, was an indication of the animals being en route to NAFLD development [30]. Azzalini et al. have shown that nose-only exposure of Zucker obese rats to cigarette smoke, which mimics SHS exposure, results in enhancement of the histological severity of NAFLD and concomitant increases in oxidative stress and hepatocellular apoptosis [28].

Recently, we have investigated the role of SHS in the development of metabolic liver disease by characterizing the global regulation of genes and molecular pathways and gene networks in mice sub-chronically exposed to SHS (four months’ exposure). Histological examination of liver tissues from SHS-exposed mice versus controls revealed significant fat accumulation (steatosis), which progressively increased as the exposed animals underwent recovery in clean air. Genome-wide gene expression analysis identified a large number of aberrantly expressed transcripts in the SHS-exposed mice upon termination of exposure (*n* = 473). The number of differentially expressed genes in the SHS-exposed mice having undergone one-month recovery in clean air remained substantially high (*n* = 222). The persistent transcriptional changes in the SHS-exposed mice predominantly affected genes and functional networks involved in lipid metabolism as well as in the regulation of the endoplasmic reticulum where manufacturing of lipids occurs. The perturbation of key lipid genes in the SHS-exposed mice is highly consistent with the progressive accumulation of fat in the corresponding animals. Our data support a role for SHS, independently of diet, in the genesis and progression of metabolic liver disease through deregulation of genes and molecular pathways and functional networks involved in lipid homeostasis. Our findings underscore how environmental carcinogens, such as SHS, in addition to cancer-causing effects, may contribute to metabolic liver disease (manuscript in preparation).

## 3. Modulation of DNA Oxidation and TET Activity by Environmental Exposures

**Figure 2:**
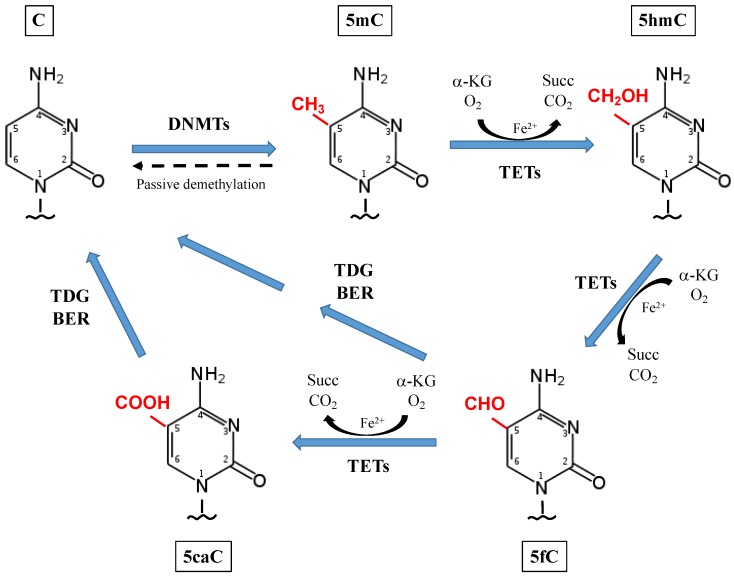
DNA methylation and demethylation in mammalian cells. DNA methylation occurs at the C5 position of cytosine (5mC), mostly in the context of CpG dinucleotides. 5mC is converted to 5-hydroxymethylcytosine (5hmC) by a DNA methylcytosine dioxygenase, belonging to the ten-eleven translocation (TET) family. TET proteins, including TET1, TET2, and TET3, can sequentially oxidize 5mC to 5hmC, to 5-formylcytosine (5fC), and finally to 5-carboxylcytosine (5caC), using oxygen, iron and α-ketoglutarate as co-factors/substrates. 5fC and 5caC are both repaired by base pair mismatch-mediated excision repair mechanisms (BER), thus resulting in conversion to cytosine, as part of an active process of demethylation. Alternatively, passive demethylation can occur during cell division.

Methylation at the C5 position of cytosine (5mC), mostly in the context of CpG dinucleotides, is the best studied epigenetic mechanism in mammalian cells, with critical functions in transcriptional regulation, development, and other biological processes [31,32,33,34,35,36]. 5mC is a stable covalent modification of the DNA; although in recent years, views on 5mC stability and persistence have drastically changed owing to new insights gained on DNA hydroxymethylation. It is now well-known that 5mC is converted to 5hmC by a DNA methylcytosine dioxygenase, belonging to the ten-eleven translocation (TET) family [37,38,39,40]. TET proteins, including TET1, TET2, and TET3, can sequentially oxidize 5mC to 5hmC, 5-formylcytosine (5fC), and finally to 5-carboxylcytosine (5caC) [41,42,43]. 5fC and 5caC are both repaired by mismatch-specific thymine DNA glycosylase (TDG) -mediated base excision repair (BER) mechanisms, thus resulting in conversion to cytosine, as part of an active process of demethylation (Figure 2) [44]. 

Conversion of 5mC to 5hmC has been associated to epigenetic reprogramming and regulation of tissue-specific genes [37,45,46]. 5hmC appears to be a prevalent epigenetic mark not just in the nuclear genome but in the mitochondrial genome as well [47,48,49]. The distribution of 5hmC within the genome also differs from that of 5mC [43,50,51,52,53,54]. Whereas 5mC is located within repetitive elements and heterochromatin, often associated to transcriptional silencing, enrichment of 5hmC is found within promoters, gene bodies, and distal *cis*-regulatory elements (i.e., enhancers), and is likely to participate in the regulation of tissue-specific expressed genes [39,54,55,56,57]. Furthermore, while the 5mC content remains constant across tissues, 5hmC is highly tissue-specific [58] and dependent on changes in the cellular state, induced by environmental and metabolic perturbations [59].

TET proteins catalyze the conversion of 5mC to 5hmC using Fe(II), O_2_ and α-ketoglutarate as co-substrates (Figure 2) [51]. α-ketoglutarate is converted to succinate during the Krebs cycle, which occurs in the matrix of mitochondria and regulates the redox state of the cells. A defective Krebs cycle is usually associated to increased oxidative stress, which causes inflammation, a hallmark of many diseases and conditions, including NASH [60]. Apart from TET enzymatic reaction, 5mC can also be converted to 5hmC, though less efficiently, via radical oxidation reactions mediated by ROS, such as hydroxyl radicals (•OH) and one-electron oxidants [41]. 

A growing number of studies has shown that environmental toxicants inducing ROS/oxidative stress affect TET protein activity and oxidation of 5mC to 5hmC, thus interfering with the epigenetic machinery and increasing susceptibility to disease (reviewed in refs [61,62,63]). Two recent studies have independently investigated the effects of redox-active quinones on TET proteins and 5hmC formation [64,65]. Both reports found that hydroquinone, a predominant metabolite of benzene and a carcinogen found in cigarette smoke and other environmental pollutants, increases TET1 activity as well as hydroxymethylation in human cells, possibly via a ROS-triggered mechanism [64,65]. Specifically, Zhao et al. reported that local enrichment of 5hmC was associated to aberrant expression of more than 3000 genes involved in a broad range of cellular functions [65]. Delatte et al. used two experimental models, namely SY5Y neuroblastoma cells treated in vitro with buthionine sulfoximine (BSO), a known inducer of oxidative stress, and double knockout mice lacking both the antioxidant enzymes *Gpx1* and *Gpx2* (*Gpx1/2 DKO* mice), to study the global patterns of 5hmC in response to oxidative stress [66]. The authors reported a global loss of 5hmC in both the in vitro and in vivo systems. However, they also identified locus-specific gain of 5hmC within coding genes and microRNAs involved in oxidative stress response pathways, as well as loss of 5hmC at genomic loci involved in the physiopathology of liver, heart, and kidney [66]. Exposure of rodents to a non-genotoxic carcinogen, phenobarbital (a known hepatocarcinogen), has also been shown to affect the patterns of 5mC/5hmC at the promoter region of a set of hepatic genes involved in xenobiotic metabolism [67]. 

A wide range of environmental carcinogens has been shown to cause perturbations in the patterns of 5hmC, often via deregulation of TET activity, with effects being strongly dependent on the intensity and duration of exposure (dose) [61,62,63,67,68,69]. Significant changes in the global level and/or distribution of 5hmC have been detected in several cancers associated with tobacco use [56,70,71] and in cells/animals exposed to a variety of environmental carcinogens/stressors [61,62,63]. The observed changes in the hydroxymethylome are often associated with chromatin remodeling and transcriptional activation [67].

## 4. The Role of 5hmC and TET Proteins in the Development of NAFLD

In recent years, there has been a growing interest in the study of epigenetic mechanisms affecting genes responsible for NAFLD development [72,73,74,75,76]. Tissue-specific epigenetic modifications, associated with the histological severity and prognosis of NAFLD, have been observed in both nuclear [77,78,79,80] and mitochondrial genomes [81]. Whereas alterations in DNA methylation, histone marks, and noncoding RNAs have been extensively investigated in fatty liver [74,75,82], data examining the impact of DNA hydroxymethylation in the initiation and progression of NAFLD remain scarce [49,83,84]. 

**Figure 3:**
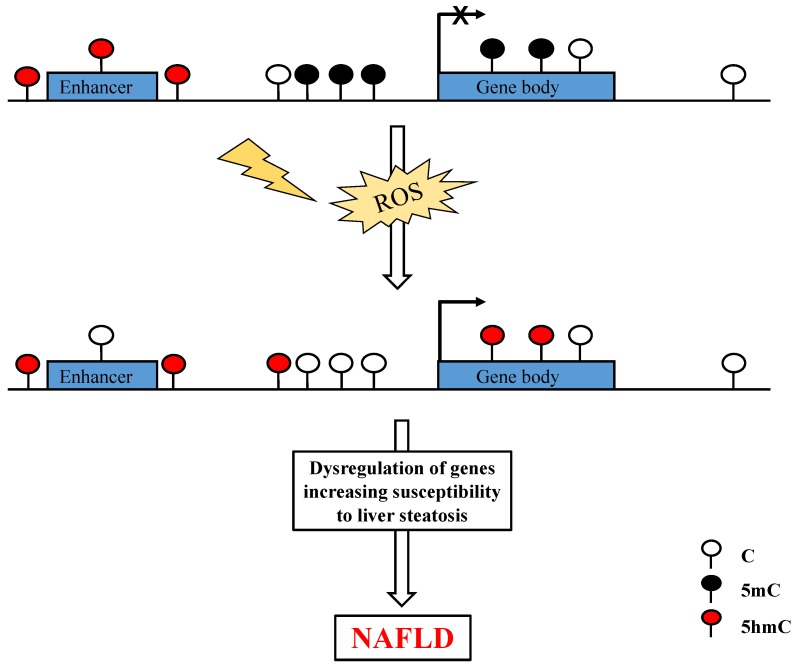
Modulation of 5hmC by oxidative stress. Enrichment of 5hmC is found within promoters, gene bodies, and distal *cis*-regulatory elements (i.e., enhancers) and is likely to participate in the regulation of tissue-specific expressed genes. Reactive oxygen species (ROS) produced by environmental toxicants and contaminants and/or as by-products of metabolism are known to accumulate within the hepatocytes of NAFLD patients. ROS and oxidative stress can alter the hydroxymethylome, thus affecting the epigenetic reprogramming and transcriptional regulation of key genes involved in lipid metabolism and/or oxidative stress response.

Pirola et al. [49] have recently analyzed the overall levels of 5hmC in fresh liver samples from NAFLD patients at different stages of the disease versus patients with near-normal liver histology. Using immuno-specific assays, the authors detected no significant differences in DNA hydroxymethylation between NAFLD samples and near-normal controls. Nevertheless, patients with NAFLD displayed a significant loss in non-nuclear 5hmC staining, probably located on mitochondria, compared to controls. Of note, the authors found a significant positive correlation between global 5hmC content and the mitochondrial DNA copy number (R = 0.50, *p* < 0.01). In addition, they observed an inverse and significant correlation with mRNA levels of the hepatic peroxisome proliferator-activated receptor gamma coactivator 1α (*PPARGC1A*) gene (R = −0.57, *p* < 0.05), a major transcription factor modulating mitochondrial biogenesis and a sensor of metabolic changes [49]. These findings are in agreement with previous data showing that NAFLD is associated with changes in *PPARGC1A* expression, mitochondrial function, and mitochondrial DNA (mtDNA) copy number [49,79,85]. Furthermore, they implied a role for 5hmC in the modulation of mitochondrial DNA methylome and transcription of genes involved in redox reactions (Figure 3), as well as in the regulation of the *PPARGC1α* gene [86]. Pirola et al. used targeted next-generation sequencing to explore the contribution of genetic variations within the three *TET* loci, of relevance to NAFLD. Analysis of missense variants in *TET1* and *TET2* revealed a putative role for the *TET1* locus in the modulation of apoptosis and liver injury in NAFLD, while the *TET2* locus is mostly involved in regulating the methylation/demethylation balance of the liver *PPARGC1α* and thus its transcription [49,86]. Of note, TET2 is the dominant TET isoform expressed in the liver [87]. 

Using experimental models of liver fibrosis (both human and rodent), Page et al. investigated the involvement of 5hmC and TET proteins during hepatic fibrogenesis [83]. The authors demonstrated that liver fibrosis is accompanied by alterations in the global patterns of 5mC/5hmC and their regulatory enzymes, which are probably indicative of genome-wide changes in gene expression [83]. Specifically, they showed increased expression of the maintenance DNA methyltransferase DNMT1 and of the de novo DNA methyltransferases DNMT3a and 3b, with concomitant reduction of TET enzymes. Next generation sequencing analysis of quiescent and activated rat hepatic stellate cells (HSCs), the key cell type responsible for the initiation and progression of liver fibrosis, identified dynamic remodeling of 5mC and 5hmC marks, genome-wide, during in vivo HSC transdifferentiation. Areas of high density of 5hmC modifications were observed particularly on chromosome 9 and were unique to activated HSCs, suggesting that site-specific hydroxymethylation plays a crucial role in fibrogenesis [83]. Unfortunately, because of the relatively poor annotation of the rat genome, a large number of differentially hydroxymethylated (and methylated) regions and their associated genes could not be identified. The mechanisms by which genome-wide alterations in the patterns of 5mC/5hmC promote changes in gene transcription during fibrogenesis are unknown, but they probably involve gene silencing by the recruitment of chromatin remodeling complexes and/or reactivation of genes by TET-induced oxidation of 5mC to 5hmC [83].

Lyall et al. used hepatocyte-like cells (HLCs) exposed to a cocktail of lactate, pyruvate, and octanoid acid (LPO) to induce steatosis, mitochondrial dysfunction, and oxidative stress, thus mimicking NAFLD initiation and progression [84]. Genome-wide profiling of 5hmC in LPO-treated and control HLCs showed no global 5hmC changes in LPO-exposed HLCs relative to the control. However, local enrichment of 5hmC was observed within the bodies of certain LPO-induced genes involved in lipid synthesis and transport, including *CYP2J2*, *HMGCS2*, *APOA4*, *APOA5*, *ACADVL*, *PCK1*, *CIDEC*, *IGFBP1*, and *PLIN2*. Because gene body hydroxymethylation is mostly associated with higher gene transcription [54], it is plausible that the intragenic enhancement of 5hmC induces up-regulation of these lipid-specific genes and, in turn, promotes the cellular steatosis observed in LPO-exposed HLCs. Enzymes involved in pathways relevant to energy metabolism were also dysregulated consistent with mitochondria dysfunction, which is a central feature of NAFLD [84]. These include isocitrate dehydrogenase 1 and 2 (IDH1 and IDH2), whose expression is known to determine the overall 5hmC content in the adult liver [88].

Altogether, these studies provide evidence of the role of DNA hydroxymethylation and TET activity, in the pathogenesis of NAFLD. Finally, it is worth mentioning that the dynamic interplay of DNA methylation/demethylation depends on the availability of substrates required for the maintenance of the epigenetic landscape and that severe/persistent fluctuations in the levels of these substrates greatly affect the patterns of epigenetic modifications. It is now known that certain metabolites produced by the gut microbiota influence the host’s long term physiology by modulating the epigenome, for example by inhibiting epigenetic modifying enzymes [89]. As a consequence, tissues exposed to high concentrations of these bacterial metabolites, such as the intestinal epithelium or the liver, may undergo epigenetic changes affecting genes involved in metabolic regulation and, in the long run, alter organ homeostasis and predispose the host to metabolic chronic disease, including NAFLD (Figure 1) [90]. One of such substances is the α-ketoglutarate, a co-factor of TET protein family members, which is produced by bacterial metabolism and can affect the host’s hydroxymethylome [91]. However, more studies are needed to further explore the crosstalk between microbiota metabolites and epigenetic machinery in chronic liver disease.

## 5. Conclusions

The prevalence of NAFLD amongst children, youth, and adult populations is increasing at an alarming rate [2,92]. Yet, the pathogenesis of this chronic liver disease is not fully understood. NAFLD is a complex and multifactorial disease. Multiple elements and conditions are now known to be involved in the progression of NAFLD, from simple steatosis and fatty liver to NASH to cirrhosis and hepatocellular carcinoma (HCC). These include diet, genetic predisposition, and gut microbiota [5,93,94]. An increasing number of studies has also provided evidence for the role of xenobiotics in the genesis of steatosis and steatohepatitis, through increased ROS production and oxidative stress [95]. Based on the current evidence, it is plausible that imbalances in the redox state of the cells, induced by exposure to a variety of pollutants, alter the hydroxymethylome, thus affecting the epigenetic reprogramming and transcriptional regulation of key genes involved in lipid metabolism or oxidative stress response (Figure 3). Aberrant expression of these genes may contribute to liver steatosis and exacerbate liver injury, thus promoting the progression to NASH. Given the reversible nature of epigenetic modifications, there is growing interest in developing epigenetic-based therapies to prevent the development of NALFD, and identifying non-invasive biomarkers to distinguish various stages of the disease.
